# Emergency hospitalization caused by non-COVID-19 respiratory diseases before and during the COVID-19 pandemic: A retrospective observational cohort study

**DOI:** 10.3389/fmed.2022.929353

**Published:** 2022-08-04

**Authors:** Qi Liu, Bingcao Lin, Changju Zhu, Jianping Hu

**Affiliations:** ^1^Department of Emergency Intensive Care Unit, The First Affiliated Hospital of Zhengzhou University, Zhengzhou, China; ^2^Department of Translational Medicine Center, The First Affiliated Hospital of Zhengzhou University, Zhengzhou, China; ^3^Department of Emergency Medicine, The First Affiliated Hospital of Zhengzhou University, Zhengzhou, China; ^4^Henan Medical Key Laboratory of Emergency and Trauma Research, The First Affiliated Hospital of Zhengzhou University, Zhengzhou, China; ^5^Department of Clinical Evaluation, Henan Medical Association, Zhengzhou, China

**Keywords:** COVID-19, emergency hospitalization, respiratory diseases, prognosis, emergency management (EM)

## Abstract

**Background:**

The coronavirus disease 2019 (COVID-19) pandemic as well as the subsequent prevention and control measures is like a quasi-experiment intervention that might have changed the features of emergency hospitalizations. Mortality is high in patient hospitalization due to emergency respiratory diseases (ERD). Therefore, we compared the characteristics of these patients before and during the pandemic. Exploring this issue might contribute to decision-making of emergency management when most of the resources and attention has been devoted to combat COVID-19.

**Methods:**

This study was a retrospective observational cohort study. All emergency hospitalizations due to ERD from January 1, 2019 to December 31, 2020 in a tertiary hospital in China were included. Data including patients’ age, sex, and clinical outcomes were extracted. Air quality was collected from the official online platform. Clinical characteristics were compared and odds ratios were calculated.

**Results:**

The ERD hospitalization rate was lower in 2020 than in 2019 (6.4 vs. 4.3%, *χ^2^* = 55.449, *P* = 0.000) with a 50.65% reduction; however, the patients were older in 2020 than in 2019 (*P* = 0.000) with a higher proportion of admission to the intensive care unit (ICU) (46 vs. 33.5%, *χ^2^* = 20.423, *P* = 0.000) and a longer ICU stay (*P* = 0.000). The overall intubation rate, hospital mortality, and rate of discharge due to ineffective treatment in 2020 were higher than those in 2019 (15.6 vs. 8%, *χ^2^* = 18.578, *P* = 0.000; 4.2 vs. 1.1%, *χ^2^* = 4.122, *P* = 0.000; 5.5 vs. 2.4%, *χ^2^* = 8.93, *P* = 0.000, respectively). The logistic regression analysis indicated hospitalizations due to ERD were mainly associated with PM2.5 and sulfur dioxide on the day, and on the 4th and 5th days before admission (*P* = 0.034 and 0.020, 0.021 and 0.000, 0.028, and 0.027, respectively) in 2019. However, in 2020, the relationship between parameters of air quality and hospitalization changed.

**Conclusion:**

The COVID-19 pandemic has changed the characteristics of emergency hospitalization due to ERD with a larger proportion of severe patients and poorer prognosis. The effect of air quality on emergencies were weakened. During the COVID-19 pandemic, it is necessary to pay more attention to the non-COVID-19 emergency patients.

## Introduction

Over the past 2 years, the coronavirus disease 2019 (COVID-19) pandemic has caused unprecedented challenges in the worldwide healthcare system and critical care medicine (1). At present, there is still no sign of the pandemic receding. To reduce the effect of COVID-19 on human, various prevention and control strategies have been adopted around the world (2). China has adopted a few control strategies aimed to keep the dynamic clearing target and has achieved an acceptable effect (3) although the control measures might result in substantial productivity losses, which account for about 2.7% of China’s annual gross domestic product (US dollar 382.29 billion) (4). In China, the public are required to adhere to some personal protection measures (PPMs) including wearing a mask, keeping proper social distance, and maintaining hand hygiene during daily life.

The mortality rate was high in emergency patient hospitalization because of respiratory diseases (5). It was shown that emergency respiratory diseases (ERD) causing emergency visits and hospitalizations were mainly affected by infection through the airway (6, 7) and poor air quality (8–11). The COVID-19 epidemic situation has improved the recognition and knowledge about respiratory diseases by the public, and simultaneous PPMs have become a habit in China. Therefore, wearing a mask and keeping social distance and hand hygiene might play an important role in preventing respiratory tract infection of the public. In addition, masks could protect people from particulate matter exposure (12) and cold air (13) because of the filtration and partition effect. In the years prior to COVID-19, few people in China have had the habit of wearing masks unless the job required it. The lifestyle change by the pandemic accompanied with the control measures is just like a quasi-experiment intervention that might have changed the features of emergency visits and hospitalizations including the emergency hospitalization due to ERD. Exploring this issue might contribute to decision-making of the emergency management while most of the resources and attention has been devoted to COVID-19. In this study, the pandemic along with the PPMs was considered as an exposure, we primarily aimed to expound the effect of this exposure on the hospitalization characteristics due to ERD pre- and post- COVID-19 pandemic. the secondary objective was to explore whether the exposure would weakened the effect of air quality on the emergency hospitalization.

## Materials and methods

### Participants

This retrospective observational cohort study was approved by the Ethics Committee of the First Affiliated Hospital of Zhengzhou University (No: 2021-KY-0587). All emergency hospitalizations because of ERD from January 1, 2019 to December 31, 2020 in the first affiliated hospital of Zhengzhou university were included in the study. Data were extracted from electronic emergency medical record system, which were searched for the hospitalized patients and then the final diagnosis of patients were checked according to the international classification of diseases one by one for the causes of emergency hospitalization in the hospital information system. The causes were firstly classified into ERD and non-ERD, and then the specified cause of emergency hospitalization due to ERD was recorded. Additionally, patients diagnosed as COVID-19 were not included in this study although COVID-19 itself was ERD, these patients were firstly managed in the fever clinic and then hospitalized in the ward for infectious diseases; the non-COVID-19 patients with fever were included when they returned to the emergency department after being eliminated by the fever clinic from the suspected cases of COVID-19 according to the China medical visit guide. Information about the included patients were extracted and stored in Excel including patients’ age, sex, ward of hospitalization, intubation or not, and clinical outcomes.

### Atmosphere air quality

In consideration of the emergency visits and hospitalizations might be affected by atmosphere air quality and climate change, we collected data of air quality in the district where the hospital was located from the official online monitoring and analysis platform in China.^1^ The key related parameters of air quality and weather included air quality index, air quality grade, particulate matter 2.5 (PM2.5), PM10, PM 25, sulfur dioxide, carbon monoxide, nitrogen dioxide, ozone, and air temperature. According to the calendar year, spring, summer, fall, and winter started on February 4th, May 6th, August 8th, and November 8th in 2019 and February 4th, May 5th, August 7th, and November 7th in 2020, respectively.

### Statistical analysis

Continuous outcomes with abnormal distribution tested by Kolmogorov-Smirnov were reported as median and interquartile range (IQR) and compared by non-parametric rank sum test. For discontinuous outcomes, the data were expressed as the number of a certain event and the proportions, and then analyzed by Chi-square test. Air quality parameters were performed regression analysis according to those on the day of hospitalization for ERD and non-hospitalization for ERD by multiple factor binary logistic regression to get the odds ratios (ORs) and confidence intervals (CIs). For the air quality parameters on the fourth, fifth, sixth, and seventh days before the day of ERD and non-ERD, regression analysis was performed in the same way to reflect the lag effect of air quality. The parameters which were found independently associated with the hospitalization for ERD were further analyzed according to the level of the parameters and the number of laging days. Statistical significance was set at a *P* value of < 0.05. The statistical softwares we employed were SPSS version 26.0 (IBM Corp., Armonk, NY) and Stata/IC 16.1 single user’s version (StataCorp LLC.,TX, United States).

## Results

### Characteristics of emergency hospitalization due to emergency respiratory diseases before and during the COVID-19 pandemic

**FIGURE 1:**
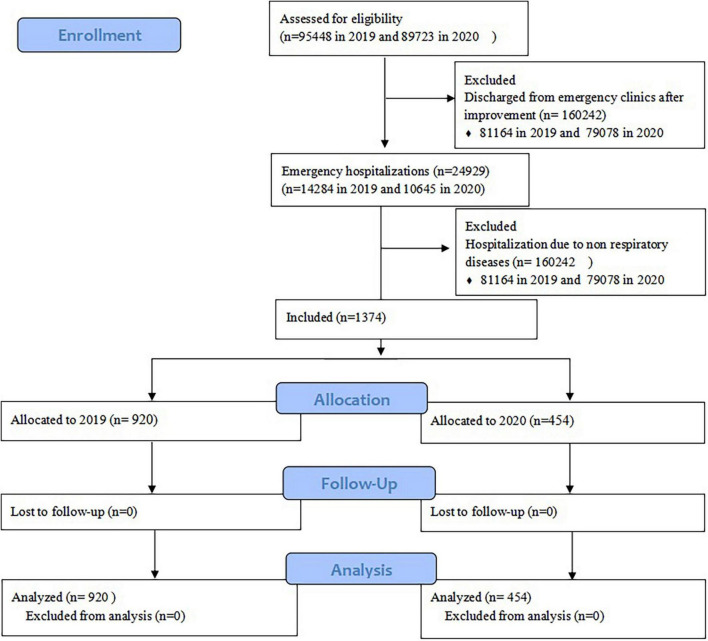
Flow diagram.

**FIGURE 2:**
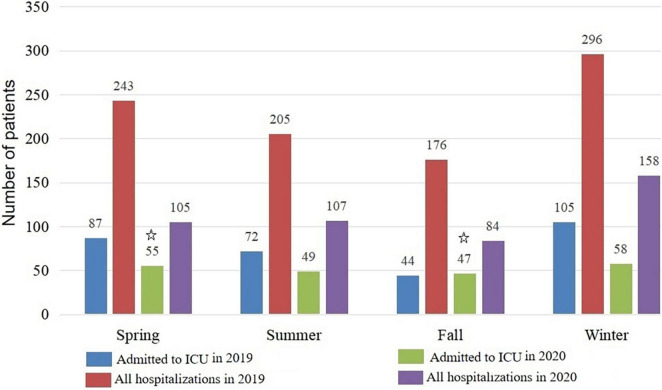
Distribution of emergency hospitalizations by season due to respiratory diseases. ICU, intensive care unit; ^
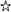
^ the ICU hospitalization rate was higher in spring and autumn of 2020 than the corresponding seasons of 2019 (55.38 vs. 35.8%, *χ^2^* = 8.343, *P* = 0.004; 55.95 vs. 25%, *χ^2^* = 23.946, *P* = 0.000, respectively).

As shown in Figure 1, in the year of 2019, before the COVID-19 pandemic, there were 95,448 patients visiting to the emergency department of the first affiliated hospital of Zhengzhou university. All cause hospitalization was 14,284, and the rate was 15.0%. There were 920 patients hospitalized due to ERD, accounting for 0.96% of the total number of emergency outpatients and 6.4% of the all cause hospitalizations. In 2020, under the COVID-19 pandemic, there were 89,723 emergency outpatients, the all cause hospitalizations and rate were 10,645 and 11.9%, respectively, there were 454 patients who were hospitalized due to ERD accounting for 0.51% of the total emergency outpatients and 4.3% of the all cause hospitalizations. The all cause and ERD hospitalization rates were lower in 2020 than those in 2019 (*χ^2^* = 387.12 and 55.449, *P* = 0.000 and *P* = 0.000, respectively). There was a 50.65% reduction in emergency hospitalization due to ERD. However, the patients were older in 2020 than those in 2019 (*P* = 0.000) with a higher rate of tracheal intubation (15.6 vs. 8%, *P* = 0.000). In 2020, the proportion of patients admitted to the intensive care unit (ICU) was higher (46 vs. 33.5%, *χ^2^* = 20.423, *P* = 0.000) with a longer ICU stay (*P* = 0.000). The survival rate (78.4 vs. 88.9%, *χ^2^* = 26.942, *P* = 0.000) in 2020 was higher than 2019 with higher hospital mortality (4.2 vs. 1.1%, *χ^2^* = 14.122, *P* = 0.000), discharge due to ineffective treatment (5.5 vs. 2.4%, *χ^2^* = 8.93, *P* = 0.000) and discharge against clinical advice (11.9 vs. 7.6%, *χ^2^* = 6.8, *P* = 0.000). There was no significant difference in the length of hospital stay between the 2 years (*P* = 0.648). The pandemic along with the PPMs reduced the proportion of community acquired pneumonia (*χ^2^* = 7.667, *P* = 0.006) and lower airway infections (*χ^2^* = 14.582, *P* = 0.000), but increased the risk of asthma attack (*χ^2^* = 5.761, *P* = 0.016), or acute respiratory failure (*χ^2^* = 30.09, *P* = 0.000), the difference in other ERDs was not significant (*P* > 0.05) (Table 1). The distribution of hospitalizations by season is shown in Figure 2. There was no significant difference in the ratio of hospitalization in each season between the 2 years, but the ICU hospitalization rate was higher in spring and autumn of 2020 than the corresponding seasons of 2019 (55.38 vs. 35.8%, *χ^2^* = 8.343, *P* = 0.004; 55.95 vs. 25%, *χ^2^* = 23.946, *P* = 0.000, respectively).

**TABLE 1:** Clinical characteristics of hospitalized patients due to ERD, air quality on the day of hospitalization.

Characteristics	Overall	Pre-pandemic (2019)	During pandemic (2020)	Z/*χ^2^*	*P*
Age, M (IQR), year	56.0 (18, 71)	51.0 (9, 70)	61.5 (37, 72)	−4.744	0.000
Male, n (%)	871 (63.4)	585 (63.6)	286 (63.0)	0.046	0.831
**Hospitalization**					
All cause hospitalization, n (%)	24,929	14,284 (15.0)	10,645 (11.9)	381.72	0.000
ERD hospitalization, n (%)	1,374 (5.51)	920 (6.4)	454 (4.3)	55.449	0.000
General ward, n (%)	857 (62.4)	612 (66.5)	245 (54.0)	20.423	0.000
ICU, n (%)	517 (37.6)	308 (33.5)	209 (46.0)	20.423	0.000
Length of ICU stay, median (IQR), day	4 (2, 8)	4 (2, 8)	5 (2, 8)	−4.946	0.000
Length of hospital stay, median (IQR), day	8.0 (5, 13)	8 (5, 12.75)	8 (5, 14)	−0.457	0.648
Tracheal intubation, n (%)	145 (10.6)	74 (8.0)	71 (15.6)	18.578	0.000
Main diagnosis of ERD, n (%)	1,374	920 (67)	454 (33)		
CAP	651 (47.4)	460 (50)	191 (42.1)	7.667	0.006
Low airway infection	124 (9)	102 (11.1)	22 (4.8)	14.582	0.000
Acute exacerbation of bronchiectasis	23 (1.7)	18 (2)	5 (1.1)	1.351	0.245
Acute attack of asthma	37 (2.7)	18 (2)	19 (4.2)	5.761	0.016
AECOPD	104 (7.6)	66 (7.2)	38 (8.4)	0.622	0.430
Pulmonary interstitial fibrosis	51 (3.7)	37 (4)	14 (3.1)	0.748	0.387
Acute respiratory failure	111 (8.1)	45 (4.9)	66 (14.5)	30.09	0.000
Pulmonary tumor	100 (7.28)	64 (6.9)	36 (7.9)	0.426	0.514
The others	173 (12.6)	110 (11.9)	63 (13.8)	1.018	0.313
**Prognosis**					
Survival, n (%)	1174 (85.4)	818 (88.9)	356 (78.4)	26.942	0.000
ICU	336 (65.0)	214 (69.5)	122 (58.4)	6.751	0.009
General ward	838 (97.7)	604 (98.6)	234 (95.5)	8.175	0.004
Hospital mortality, n (%)	29 (2.1)	10 (1.1)	19 (4.2)	14.122	0.000
ICU	24 (4.6)	9 (2.9)	15 (7.2)	5.092	0.024
General ward	5 (0.6)	1 (0.2)	4 (1.6)	4.225	0.040
DDIT, n (%)	47 (3.4)	22 (2.4)	25 (5.5)	8.930	0.003
ICU	43 (8.3)	21 (6.8)	22 (10.5)	2.245	0.134
General ward	4 (0.5)	1 (0.2)	3 (1.3)	2.264	0.132
DACA, n (%)	124 (9)	70 (7.6)	54 (11.9)	6.800	0.009
ICU	114 (22.1)	64 (20.8)	50 (23.9)	0.716	0.397
General ward	10 (1.2)	6 (1.0)	4 (1.6)	0.204	0.652
**Air quality on the day of emergency, median (IQR), mcg/m^3^**					
PM2.5	44.0 (30, 80)	44.5 (31, 83)	43 (28, 73.3)	−1.886	0.059
PM10	92.0 (69, 131)	93.5 (70.3, 136)	90 (68, 123)	−2.380	0.017
SO_2_	9.0 (7, 12)	10 (7, 13)	9 (7, 12)	−1.700	0.089
CO	0.8 (0.6, 1.1)	0.8 (0.7, 1.2)	0.8 (0.6, 1.0)	−4.691	0.000
NO_2_	43.0 (32, 57)	44 (34, 60)	41 (29, 54)	−4.431	0.000
O_3_	93.0 (54, 143)	90 (54, 141.5)	100 (54, 146.25)	−0.887	0.375
AQ index	98.0 (75, 134)	102 (75, 135)	94 (75, 130)	−1.631	0.103
AQ grade^¥^, M (IQR)	2.0 (2, 3)	3.0 (2, 3)	2.0 (2, 3)	−2.422	0.015
Temperature, M (IQR), °C	14.1 (4.4, 23.90)	13.7 (4.3, 23.4)	14.5 (4.8, 24.9)	−0.614	0.539

PM2.5, particulate matter 2.5; PM10, particulate matter 10; PM25, particulate matter 25; SO_2_, sulfur dioxide; CO, carbon monoxide; NO_2_, nitrogen dioxide; AT, air temperature; IQR, interquartile range; ICU, intensive care unit; DDIT, discharge due to ineffective treatment; DACA, discharge against clinical advice; ^¥^AQ grade: 1 = excellent, 2 = good, 3 = mild pollution, 4 = moderate pollution, 5 = heavy pollution, 6 = severe pollution.

### Effect of air quality on the emergency hospitalization due to emergency respiratory diseases

**FIGURE 3:**
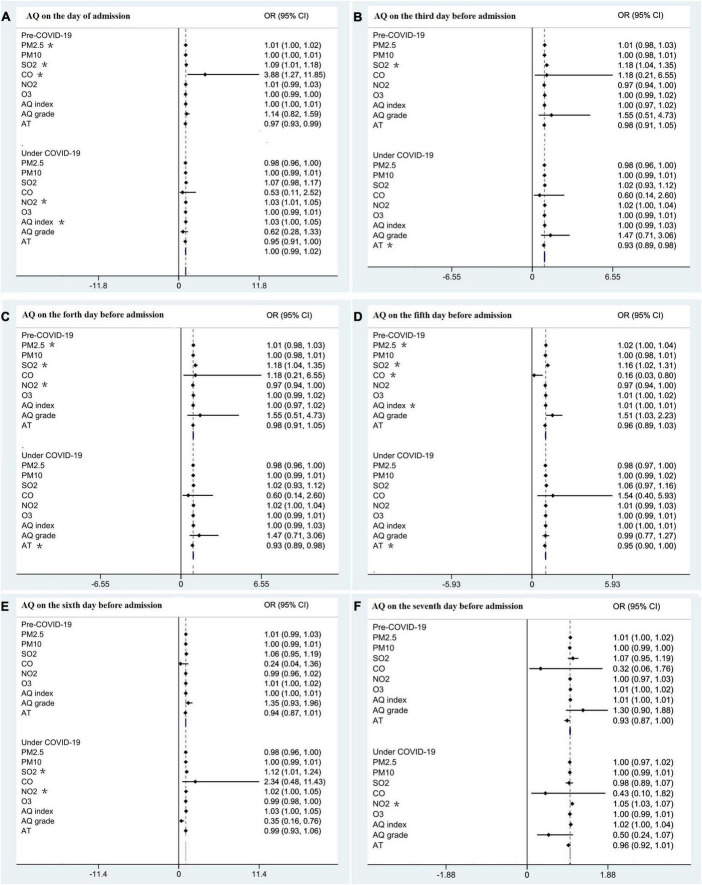
The effect of AQ on the admission pre and under COVID-19. **(A)** AQ on the day of admission. **(B)** AQ on the third before admission. **(C)** AQ on the fourth before admission. **(D)** AQ on the fifth before admission. **(E)** AQ on the sixth before admission. **(F)** AQ on the seventh before admission. AQ, air quality; OR, odds ratio; CI, confidence interval; PM2.5, particulate matter 2.5; PM10, particulate matter 10; PM25, particulate matter 25; SO_2_, sulfur dioxide; CO, carbon monoxide; NO_2_, nitrogen dioxide; AQ grade: 1 = excellent, 2 = good, 3 = mild pollution, 4 = moderate pollution, 5 = heavy pollution 6 = severe pollution; *indicates a significant result. AT, air temperature.

Between the 2 years, most parameters of air quality on the day of hospitalization were non-significant except for PM10 (median, 93.57; IQR 10.3–136 vs. median, 90; IQR 18–123, *P* = 0.017), carbon monoxide (median, 0.8; IQR 0.7–1.2 vs. median, 0.8; IQR 0.6–1.1, *P* = 0.000), nitrogen dioxide (median, 44; IQR 34–60 vs. median, 41; IQR 29–54, *P* = 0.000) (Table 1). The multivariate regression analysis indicated the hospitalizations due to ERD were mainly associated with PM2.5 and sulfur dioxide on the day of admission and on the 4th and 5th days before the admission (*P* = 0.034 and 0.020, 0.021 and 0.000, 0.028, and 0.027, respectively) in 2019. However, in 2020, the relationship between parameters of air quality and the hospitalization changed. There were only a few specific parameter of air quality that could be considered as potential risk factors of hospitalization. The effect of air quality on hospitalization was not dramatically affected by the season in both 2019 and 2020 (Table 2). As shown in Figure 3, the emergency hospitalization was affected by air quality (mainly PM2.5,SO_2_,NO_2_) on the fourth day (Figure 3C), on the fifth day (Figure 3D) before the admission pre-COVID-19, which reflected the significant hysteresis effect, whereas the effect was weakened during COVID-19 pandemic.

**TABLE 2:** Multiple factor binary logistic regression investigating independent predictors of admission due to ERD.

2019	AQ on the day of admission				AQ on the 4th day before admission				AQ on the 5th day before admission				AQ on the 6th day before admission				AQ on the 7th day before admission			
Variable	OR	LI	UI	*P*	OR	LI	UI	*P*	OR	LI	UI	*P*	OR	LI	UI	*P*	OR	LI	UI	*P*
PM2.5	1.010	1.001	1.019	0.034	1.022	1.003	1.042	0.021	1.023	1.002	1.044	0.028	1.009	0.990	1.029	0.337	1.008	0.999	1.016	0.091
PM10	1.005	0.999	1.012	0.123	0.993	0.981	1.004	0.200	0.996	0.984	1.009	0.587	0.999	0.988	1.011	0.931	0.995	0.986	1.005	0.346
SO_2_	1.094	1.014	1.179	0.020	1.397	1.204	1.621	0.000	1.156	1.016	1.314	0.027	1.063	0.949	1.190	0.292	1.066	0.952	1.193	0.270
CO	3.877	1.269	11.849	0.017	0.682	0.120	3.891	0.667	0.155	0.030	0.804	0.026	0.240	0.042	1.364	0.107	0.317	0.057	1.756	0.188
NO_2_	1.013	0.994	1.032	0.175	0.949	0.919	0.980	0.001	0.974	0.945	1.004	0.090	0.992	0.963	1.022	0.613	1.001	0.971	1.030	0.973
O_3_	0.995	0.990	1.001	0.076	0.998	0.989	1.009	0.767	1.008	0.998	1.019	0.098	1.007	0.997	1.017	0.152	1.009	0.999	1.019	0.079
AQ index	1.004	0.998	1.011	0.208	1.004	0.997	1.011	0.250	1.007	0.999	1.014	0.076	1.005	0.998	1.011	0.195	1.006	0.999	1.014	0.096
AQ grade	1.142	0.819	1.593	0.433	1.188	0.826	1.707	0.353	1.514	1.027	2.232	0.036	1.353	0.932	1.963	0.112	1.303	0.904	1.878	0.155
AT	0.968	0.932	0.994	0.963	1.055	0.978	1.138	0.169	0.959	0.891	1.033	0.272	0.936	0.870	1.008	0.079	0.934	0.868	1.005	0.069
**Seasons (Summer as reference)**																				
Spring	1.524	0.558	4.164	0.411	2.059	0.784	5.412	0.143	2.112	0.804	5.552	0.129	2.152	0.819	5.657	0.120	2.173	0.826	5.711	0.116
Autumn	0.594	0.248	1.424	0.243	0.797	0.348	1.825	0.592	0.819	0.358	1.875	0.636	0.764	0.338	1.729	0.519	0.769	0.340	1.738	0.527
Winter	1.300	0.519	3.254	0.576	2.186	0.863	5.532	0.099	2.247	0.888	5.689	0.088	2.605	0.992	6.838	0.052	2.619	0.998	6.876	0.051

**2020**	**AQ on the day of admission**				**AQ on the 4th day before admission**				**AQ on the 5th day before admission**				**AQ on the 6th day before admission**				**AQ on the 7th day before admission**			
**Variable**	**OR**	**LI**	**UI**	***P***	**OR**	**LI**	**UI**	***P***	**OR**	**LI**	**UI**	***P***	**OR**	**LI**	**UI**	***P***	**OR**	**LI**	**UI**	***P***

PM2.5	0.984	0.962	1.005	0.133	1.006	1.000	1.012	0.051	0.984	0.968	1.001	0.072	0.983	0.962	1.005	0.130	0.995	0.974	1.016	0.640
PM10	0.999	0.988	1.010	0.807	1.007	0.995	1.019	0.235	1.005	0.994	1.016	0.395	1.000	0.988	1.011	0.955	0.997	0.986	1.008	0.583
SO_2_	1.071	0.976	1.175	0.150	1.028	0.937	1.127	0.562	1.061	0.968	1.162	0.208	1.120	1.012	1.240	0.028	0.977	0.894	1.067	0.606
CO	0.534	0.113	2.516	0.428	1.619	0.326	8.032	0.555	1.541	0.400	5.933	0.530	2.339	0.479	11.428	0.294	0.430	0.102	1.819	0.251
NO_2_	1.031	1.008	1.055	0.007	1.015	0.994	1.037	0.156	1.013	0.993	1.034	0.195	1.024	1.002	1.047	0.031	1.051	1.028	1.074	0.000
O_3_	1.000	0.990	1.010	0.996	1.000	0.990	1.010	0.990	1.000	0.993	1.008	0.919	0.991	0.981	1.002	0.101	0.998	0.988	1.007	0.636
AQ index	1.027	1.005	1.050	0.017	1.021	0.999	1.043	0.057	1.001	0.996	1.007	0.583	1.026	1.003	1.050	0.026	1.020	0.999	1.042	0.068
AQ grade	0.616	0.285	1.332	0.219	0.485	0.225	1.046	0.065	0.990	0.771	1.271	0.937	0.349	0.161	0.757	0.008	0.504	0.238	1.070	0.075
AT	0.953	0.909	0.998	0.041	0.920	0.865	0.979	0.008	0.947	0.900	0.996	0.034	0.992	0.930	1.058	0.806	0.964	0.918	1.013	0.144
**Seasons (Summer as reference)**																				
Spring	1.076	0.617	1.877	0.796	0.519	0.216	1.243	0.141	1.166	0.666	2.040	0.591	0.662	0.263	1.666	0.381	1.373	0.780	2.414	0.272
Autumn	0.861	0.490	1.514	0.603	0.523	0.270	1.013	0.055	0.877	0.499	1.544	0.650	0.414	0.211	0.814	0.011	0.930	0.529	1.633	0.800
Winter	2.789	1.498	5.194	0.001	0.974	0.296	3.209	0.965	2.676	1.449	4.941	0.002	0.590	0.174	2.001	0.397	2.676	1.464	4.894	0.001

ERD, emergency respiratory diseases; OR, odd ratio; LI, lower interval of 95% confidence interval; UI, upper interval of 95% confidence interval; AQ grade: air quality grade, 1 = Excellent, 2 = good, 3 = Mild pollution, 4 = moderate pollution, 5 = Severe pollution, 6 = Severe pollution; AT, Air temperature.

### The laging effect of PM2.5 on hospitalization due to emergency respiratory diseases

As shown in Table 3, the hysteresis of PM2.5 on hospitalizations owing to ERD was analyzed further according to the concentration grade of PM2.5. The ORs of hospitalizations owing to ERD increased with the PM2.5 concentration grade, especially on the day and the laging 5th day in 2019 and on the day and the laging 1st, 2nd, and 6th days in 2020.

**TABLE 3:** Hysteresis effect of different concentration grade of PM2.5.

Hysteresis (day)	PM2.5 ≤ 50	50 < PM2.5 ≤ 100	PM2.5 > 100
	OR (LI, UI)	OR (LI, UI)	OR (LI, UI)
**2019**			
0	0.680 (0.226, 2.051)	0.684 (0.194, 2.415)	0.841 (0.176, 4.027)
1	1.405 (0.589, 3.352)	2.822 (0.887, 8.977)	1.744 (0.499, 6.098)
2	0.889 (0.318, 2.487)	0.919 (0.276, 3.058)	0.442 (0.116, 1.682)
3	0.774 (0.281, 2.133)	1.320 (0.401, 4.342)	0.989 (0.248, 3.946)
4	0.368 (0.106, 1.276)	0.895 (0.217, 3.693)	0.590 (0.128, 2.727)
5	0.980 (0.383, 2.508)	1.359 (0.437, 4.230)	1.882 (0.417, 8.502)
6	1.336 (0.535, 3.336)	1.131 (0.384, 3.332)	0.875 (0.230, 3.327)
7	1.112 (0.395, 3.134)	0.606 (0.199, 1.842)	0.811 (0.188, 3.494)
**2020**			
0	1.664 (0.878, 3.157)	2.014 (0.953, 4.254)	3.115 (1.137, 8.536)
1	0.963 (0.500, 1.855)	1.197 (0.556, 2.579)	2.726 (0.918, 8.091)
2	0.816 (0.414, 1.608)	0.833 (0.374, 1.859)	2.057 (0.631, 6.703)
3	0.888 (0.439, 1.796)	1.021 (0.449, 2.321)	0.973 (0.339, 2.790)
4	1.135 (0.553, 2.329)	0.645 (0.282, 1.475)	0.362 (0.127, 1.034)
5	1.052 (0.526, 2.101)	0.967 (0.436, 2.145)	0.871 (0.303, 2.502)
6	0.969 (0.488, 1.925)	1.034 (0.462, 2.311)	1.619 (0.538, 4.876)
7	0.965 (0.496, 1.877)	1.108 (0.498, 2467)	1.051 (0.527, 4.326)

PM2.5, particulate matter 2.5; OR, odd ratio; LI, lower interval of 95% confidence interval; UI, upper interval of 95% confidence interval.

## Discussion

In this study, we found that the COVID-19 pandemic reduced hospitalization due to ERD, but the proportion of critical patients was higher with poorer prognosis than that in the pre-pandemic era. Before COVID-19, hospitalizations due to ERD were affected by air quality while this effect might be weakened under the pandemic of COVID-19 with a larger proportion of severe patients with poorer prognosis.

The COVID-19 pandemic causes various impacts on human activity including the emergency visit and hospitalization (14–16), this phenomenon was reported in the other respiratory infectious diseases included severe acute respiratory syndrome (17), middle east respiratory syndrome coronavirus (18), and the novel influenza A (19). Therefore, the COVID-19 pandemic might change the characteristics of emergency hospitalization due to ERD, it is necessary to identify these changes and focus more on improving the care of non-COVID-19 emergency patients during the current pandemic because the mortality was high for emergency visits due to ERD (5). In this study, we found that the number of emergency visits and hospitalization decreased sharply, which was similar with the United States (20, 21), although the epidemic control measures did not affect patients’ convenience of emergency visits. There was a 5% reduction in the number of emergency outpatient visits, a 25.5% reduction in emergency hospitalizations, and a 50.65% reduction in emergency hospitalization due to ERD, which was backed by another study (14).

This downward trend was also found in the studies on general surgical emergencies (22–24), neurological diseases (25), and serious cardiovascular events (26). This might be partially due to patients with mild discomfort avoiding the hospital for fear of being infected with COVID-19 (27). As shown in Table 1, during the pandemic, patients who were hospitalized emergently were older with a higher chance for ICU admission, a higher chance of intubation, and a longer ICU stay, which implied the patients suffered from more critical diseases. After the pandemic outbreak, the survival rate was lower and the mortality was higher in both the general ward and the ICU than those before the pandemic, which denoted a poor prognosis. This was consistent with the findings in neurological diseases (25), and out-of-hospital arrest patients (with lower rate of successful resuscitation, and higher mortality) (28). Additionally, the pandemic along with the PPMs have reduced the proportion of community acquired pneumonia and lower airway infections, but increased the risk of asthma attack, or acute respiratory failure, which might be caused by the delay to hospital due to patients’ fear of infection. These findings emphasize that it is also necessary to pay more attention to the non-COVID-19 emergency patients and ensure adequate medical personnel under this lengthy pandemic when people are involved in combating the pandemic. The hospital mortality was lower than the reported 30 day mortality (12.5%) (5), which might be attributed to traditional Chinese customs, that is, family members of the patients are unwilling to accept their relatives’ decease in hospital and would choose to take the patients home when the rescue is ineffective, and the end of life is near. We classified this portion of patients into “discharge due to ineffective treatment,” who would be clinically deceased in a very short time.

This study indicated that poor air quality prior to the COVID-19 pandemic increased the risk of emergency hospitalization for respiratory diseases and there was a certain hysteresis effect, which was consistent with the research results of a developed city, Hangzhou, in China (8). PM2.5 is particularly important among air quality indicators, as it increases the number of hospitalizations on the day and extends the laging-effect to 4 ∼ 5 days, which is longer than another study in Beijing (11). Additionally, we also found that the other gaseous pollutants such as sulfur dioxide and carbon monoxide also have an important impact on the emergency hospitalization caused by respiratory diseases. It has been stated that exposure to air contaminants provokes inflammatory reactions, disrupts the human immune system, and increases the expression of receptors that favors viruses entering the respiratory system (29). The impact of air quality on hospitalization was not different in different seasons. Therefore, the number of inpatients in different seasons is mainly related to temperature because the four seasons are distinct in our city. The outdoor temperature difference between winter and summer is about 45°C.

During the COVID-19 pandemic, the effect of air quality on emergency hospitalization was weakened, which may be related to the improvement of hygiene habits, longer social distance, and the improvement of air quality itself. In most countries, wearing a mask was recommended by public health authorities during the pandemic and the proportion of mask usage increased remarkably, which was illustrated by large consumption of masks and the extraordinarily prosperous mask technology (30), although the public in different countries have varied attitudes toward wearing masks (31). It was shown that wearing a mask could prevent the public population from COVID-19 contamination albeit robust randomized controlled trials were still needed (32). For a country with a large population, it is prone to population gathering and easy to cause disease transmission among crowds. As a result, stricter mask-related policies were adopted in China, especially when people appeared in public places. The popularization of health knowledge in the public has improved the performance of hand hygiene (33) with higher compliance (34). Wearing facemasks and keeping good hand hygiene could prevent microorganism transmission (not limited to COVID-19) (35). Keeping longer social distance further decreased the risk of exposure considering the propagation of infectious pathogens depended on the number and types of touch between contagious and susceptible hosts (36). According to the findings, PPMs might be useful in our daily life to prevent exacerbation of respiratory diseases, and may be meaningful for the older patients with underlying respiratory system diseases. One fact that has to be faced is that the effect of PPMs was affected to some extent by the adherence to PPMs based on different socio-culture and air quality, which should be considered when the findings of this part were adopted. Additionally, the improvement of air quality itself also weakened the effect of air quality on hospitalization due to ERD. According to the needs of epidemic prevention and control, the government has restricted some industrial production, which has changed the air quality of our country to a certain extent (37).

There were some limitations in this study. First, the shortcomings arose from the method of the observation research itself. Second, fever clinics may divert some emergency patients with respiratory diseases although non-COVID-19 patients with fever still need to return to the emergency after being checked by the fever clinic according to the China medical visit guide. Third, ERD conditions were defined with ICD-10 diagnosis but discrepancy might exist during the coding practices; Forth, the air quality was not completely same in the 2 years. Part of the air quality parameters were better in 2020 and might weaken the effect of air quality on acute hospitalizations due to ERD in a certain extent and even cause a bias. Finally, this was a single center study and the data only reflected the character of emergency hospitalizations in one local place, which indicated that large and multi-center trials are still needed.

In conclusion, the COVID-19 pandemic has changed the characteristics of emergency hospitalization due to ERD with a larger proportion of severe patients and poorer prognosis. The effect of air quality on emergency were weakened. During the COVID-19 pandemic, it is necessary to pay more attention to the non-COVID-19 emergency patients and to ensure adequate medical personnel. Large and multi-center trials are still needed to confirm the findings of this study.

## Data availability statement

The original contributions presented in this study are included in the article/supplementary material, further inquiries can be directed to the corresponding authors.

## Ethics statement

The studies involving human participants were reviewed and approved by the Ethics Committee of the First Affiliated Hospital of Zhengzhou University (2022-KY-0356-002). Written informed consent for participation was not required for this study in accordance with the national legislation and the institutional requirements.

## Author contributions

QL and JH conceived and designed the study, explained the results, and revised the manuscript. QL, BL, and CZ conducted the study, collected and analyzed the data, and drafted the manuscript. All authors reviewed and revised the manuscript.

## Footnotes

1
https://www.aqistudy.cn/
